# The Role of Dopamine Neurotransmitters in Neurological Diseases: New Sight

**DOI:** 10.3390/ijms25147529

**Published:** 2024-07-09

**Authors:** Yingfang Tian

**Affiliations:** 1 Key Laboratory of Modern Teaching Technology, Ministry of Education, Shaanxi Normal University, Xi’an 710062, China; yingfang_tian@snnu.edu.cn; 2 College of Life Sciences, Shaanxi Normal University, Xi’an 710119, China

Dopamine (DA) is one of the most important catecholamine neurotransmitters in the central nervous system. DA is synthesized through a restricted population of cells, which are mainly located in the ventral midbrain, and the projections arising from these brain areas constitute the fundamental framework of the dopaminergic system. Via different receptors and/or circuitries, DA implicates the regulation of many functions in the brain, such as motor control, motivation, reward, emotion, feeding, and endocrine functions. Therefore, dysfunctions in the dopaminergic system are related to a plethora of mental and physical diseases, such as Parkinson’s disease (PD), schizophrenia, Tourette’s syndrome, drug addiction, learning and memory deficits, autism, and attention deficit hyperactivity syndrome [1,2,3]. Thus, there is an increasing need to elucidate the role of DA neurotransmitters and the dopaminergic system in neurological diseases and develop effective medications or therapy strategies for treating DA-related disorders.

DA modulates structure and function in the target brain region. DA inhibits excitatory synaptic transmission in the anterior cingulate cortex (ACC), a brain region involved in acute and chronic pain. It is necessary to detect how DA modulates inhibitory synaptic transmission in the ACC and its alteration of the excitatory/inhibitory (E/I) balance during inflammation pain. It is suggested that complete Freund’s adjuvant (CFA)-induced inflammation and DA do not significantly affect the E/I balance in ACC neurons, but activity-dependent changes may be observed in the DA modulation of presynaptic glutamate release in the presence of inflammation [4]. A review also proposes DA as a model not only of neurotransmission but also of neuromodulation capable of modifying neuronal architecture. These modifications lead to synaptic plasticity and finally to morphological and functional changes, starting with maladaptive neuromodulation and ending in neurodegeneration [2].

Since DA acts as a powerful regulator of different aspects of brain function, the dopaminergic system would be an attractive therapeutic target for these diseases. A review proposed a combination therapy strategy for PD based on DA receptor agonists plus tyrosine hydroxylase (TH) inhibitors. TH inhibitors can protect the survival of remaining dopaminergic neurons, whereas DA receptor agonists activate post-synaptic DA receptors to alleviate symptoms in PD patients. This multi-drug cocktail may represent a novel strategy to protect against progressive dopaminergic neurodegeneration and alleviate PD disease progression [3]. The dopamine transporter (DAT) plays an important role in the modulation of DA neurotransmission through the reuptake of extracellular DA. S-CE-123 is a novel DAT inhibitor. The combination of improved BBB transport and higher affinity for DAT makes S-CE-123 a promising candidate for the treatment of cognitive decline [5]. Fatty acid-binding protein 3 (FABP3) specifically binds to the dopamine D2 receptor (D2R), and plays an essential role in D2R functions. As a newly developed FABP3 inhibitor, MF1 treatment suppresses D2R/FABP3 signaling, thereby preventing nicotine-induced conditioned place preference. Hence, MF1 can be used as a novel drug to block addiction to nicotine and other drugs by inhibiting the dopaminergic signaling [6].

Dopaminergic neurons are susceptible to inflammation, which is a recognized risk factor for neuronal malfunction and cell death in major neurodegenerative diseases. Bilirubin, a heme metabolite, has been shown to possess important anti-inflammatory activity. Thus, it seems to be an endogenous candidate biomolecule for improving or preventing DA-related neurological dysfunctions, supporting its potential therapeutic usage. A review summarizes the evidence linking dopaminergic neurons, inflammation, and bilirubin as an anti-inflammatory unit and presents the hypotheses of anti-inflammatory strategies aimed at preserving dopamine homeostasis with bilirubin included [1].

Although dopaminergic cells are mainly located in the ventral midbrain, the presence of dopaminergic cells in the median raphe region (MRR) has been confirmed in both mouse and human samples. These dopaminergic neurons in the MRR do not influence locomotion, anxiety, or memory; however, manipulation of these cells elicits a specific social response [7].

Overall, there are four studies and three reviews presented in this Special Issue that report new research progress and reviews concerning the physiologic DA function and the pathological trajectories in DA-related neurological diseases. These will help to understand the mechanism and pave the path for future work towards developing efficient therapies against DA-related disorders.
